# Effects of Plasma Membrane Cholesterol Level and Cytoskeleton F-Actin on Cell Protrusion Mechanics

**DOI:** 10.1371/journal.pone.0057147

**Published:** 2013-02-22

**Authors:** Nima Khatibzadeh, Alexander A. Spector, William E. Brownell, Bahman Anvari

**Affiliations:** 1 Department of Mechanical Engineering, University of California Riverside, Riverside, California, United States of America; 2 Department of Biomedical Engineering, Johns Hopkins University, Baltimore, Maryland, United States of America; 3 Bobby R. Alford Department of Otolaryngology-Head and Neck Surgery, Baylor College of Medicine, Houston, Texas, United States of America; 4 Department of Bioengineering, University of California, Riverside, California, United States of America; University of California, Berkeley, United States of America

## Abstract

Protrusions are deformations that form at the surface of living cells during biological activities such as cell migration. Using combined optical tweezers and fluorescent microscopy, we quantified the mechanical properties of protrusions in adherent human embryonic kidney cells in response to application of an external force at the cell surface. The mechanical properties of protrusions were analyzed by obtaining the associated force-length plots during protrusion formation, and force relaxation at constant length. Protrusion mechanics were interpretable by a standard linear solid (Kelvin) model, consisting of two stiffness parameters, *k*_0_ and *k*_1_ (with *k*_0_>*k*_1_), and a viscous coefficient. While both stiffness parameters contribute to the time-dependant mechanical behavior of the protrusions, *k*_0_ and *k*_1_ in particular dominated the early and late stages of the protrusion formation and elongation process, respectively. Lowering the membrane cholesterol content by 25% increased the *k*_0_ stiffness by 74%, and shortened the protrusion length by almost half. Enhancement of membrane cholesterol content by nearly two-fold increased the protrusion length by 30%, and decreased the *k*_0_ stiffness by nearly two-and-half-fold as compared with control cells. Cytoskeleton integrity was found to make a major contribution to protrusion mechanics as evidenced by the effects of F-actin disruption on the resulting mechanical parameters. Viscoelastic behavior of protrusions was further characterized by hysteresis and force relaxation after formation. The results of this study elucidate the coordination of plasma membrane composition and cytoskeleton during protrusion formation.

## Introduction

Cellular protrusions are deformations that form at the surface of living cells under certain biological conditions. Protrusions can occur during cell migration at the leading edge of the cell in form of lamellipodia and filopodia [1]–[3]. Another example of protrusions, induced during inflammation, is mediated by receptor-ligand attachments and formed at the surface of leukocytes rolling over endothelium [4]–[7]. Protrusions involve the cell plasma membrane and underlying cytoskeleton, with the interplay between these two cellular components essential in protrusion formation [8], and a determining factor for changes in protrusion phenotypes [9], [10].

Changes in membrane composition and cytoskeleton physical properties are reported to influence the behavior of cell protrusions. Depletion of membrane cholesterol inhibited the formation of microtubule-based protrusions, presumably due to changes in membrane mechanical properties upon cholesterol depletion [11]. High levels of membrane cholesterol in polymorphonuclear leukocytes attenuated the pseudopodia retraction in response to shear flows, indicating a link between plasma membrane fluidity and cellular mechanotransduction [12]. Parallel to the effects of membrane biophysical properties, cytoskeletal mechanics also have associated effects on protrusions. Cell cortical tension limited the lamellipodia outgrowth in adherent Walker carcinosarcoma cells, and acted as a determining factor for the switch between bleb and lamellipodia types of protrusion [2].

While these studies indicate the important role of plasma membrane and cytoskeleton on cell protrusion extension and retraction, the contributions of these two cellular components in protrusions have not been well established. To this end, herein, we present a quantitative investigation of the effects of plasma membrane composition and cytoskeleton integrity on mechanical properties of cellular protrusions. Protrusions were formed by application of an external force on the cell surface with an optically-trapped microsphere. We used human embryonic kidney (HEK) cells whose plasma membrane mechanical properties have been recently studied, and correlated to membrane cholesterol content by this group [13]. To discern the role of membrane composition on cell protrusions, we varied the membrane cholesterol content of HEK cells and quantified the protrusion mechanical properties using optical tweezers. To establish the role of cytoskeleton on protrusion mechanics, we repeated experiments on cholesterol enriched and cholesterol depleted cells after disruption of cytoskeleton F-actin. Relative contributions of the plasma membrane and cytoskeleton in protrusion formation and mechanics were studied through comparing their various quantified metrics under different cholesterol manipulation conditions for cells with intact and disrupted F-actin.

## Materials and Methods

### Cell Culture

Human embryonic kidney (HEK293) cells (ATCC, CRL-1573) were seeded in Dulbecco's modified eagle medium (DMEM, Gibco) with 10% fetal bovine serum (FBS, Invitrogen) and 1% Penicillin/Streptomycin (Gibco). Cells were incubated in an air jacketed CO_2_ incubator (NuAire) at 37°C with 5% CO_2_. When the cells were 70–80% confluent, they were passaged into glass bottom poly-d-lysine coated MatTek plates (P35GC-1.0-14C). Cells of medium size (≈10–20 µm diameter) were selected for measurements if they were firmly attached to the bottom of the Petri dish. All experiments were performed within 30 minutes of removal of the cells from the incubator.

### Cell membrane cholesterol manipulation

A commonly used method to modify the membrane cholesterol content is incubation of cells with cyclodextrins [14]. Cells were incubated in DMEM containing 5 mM methyl-β-cyclodextrin (MβCD) (Sigma) to reduce the membrane cholesterol content. We used water-soluble cholesterol with MβCD (51 mg cholesterol per gram of cholesterol+MβCD) (Sigma) for cholesterol enrichment. Cells were incubated in DMEM containing 5 mM water-soluble cholesterol (at 5 mM MβCD concentration) to enrich the membrane cholesterol content. Incubation time was 30 minutes at 37°C and 5% CO_2_ in both cholesterol depletion and cholesterol enrichment experiments [15].

### Cell membrane cholesterol quantification

We assumed the membrane cholesterol concentrations for each cholesterol modulation condition to be the same as those reported in our previous report [16]. In that study, membrane cholesterol content for control and cholesterol manipulated HEK cells was quantified using the Amplex Red cholesterol assay. Briefly, this colorimetric assay is based on the reaction of cholesterol with cholesterol oxidase to yield H_2_O_2_ which can be detected using the Amplex Red reagent. The membrane cholesterol concentration for normal (control) HEK cells is 7.5±0.8 pmol/µg protein. The membrane cholesterol concentration in cholesterol depleted cells, based on the use of 5 mM MβCD concentration, is 5.7±0.8 pmol/µg protein. In the case of cholesterol enriched cells, the membrane cholesterol concentration, based on the use of 5 mM water-soluble cholesterol at 5 mM MβCD concentration, is 17.3±0.6 pmol/µg protein.

### Cytoskeletal F-actin disruption

We used Latrunculin-A dissolved in dimethyl sulfoxide (DMSO) (EMD Chemicals, Gibbstown, NJ) as a reagent that inhibits F-actin polymerization. Specifically a 1∶1 molar complex between Latrunculin-A and G-actin forms, preventing the G-actin from repolymerization into filaments [17], [18]. It is reported that treatment of cells with Latrunculin-A results in decoupling of the cytoskeleton actin from the plasma membrane; for example, transmembrane proteins such as integrins and cadherins lose their connections to F-actin [19]. F-actin disruption was performed after each type of cholesterol modulation, and prior to the protrusion formation experiments. Specifically, cells were incubated in DMEM, and Latrunculin-A solution was then added to DMEM to a final concentration of 2 µM. Cells were incubated in Latrunculin-A containing medium for ≈10 minutes at 37°C and 5% CO_2_, and the medium was then replaced by DMEM at 37°C prior to protrusion formation experiments. There was no observable evidence of damage to cells due to DMSO application.

### Optical tweezers setup, force calibration, and fluorescence microscopy

**Figure 1:**
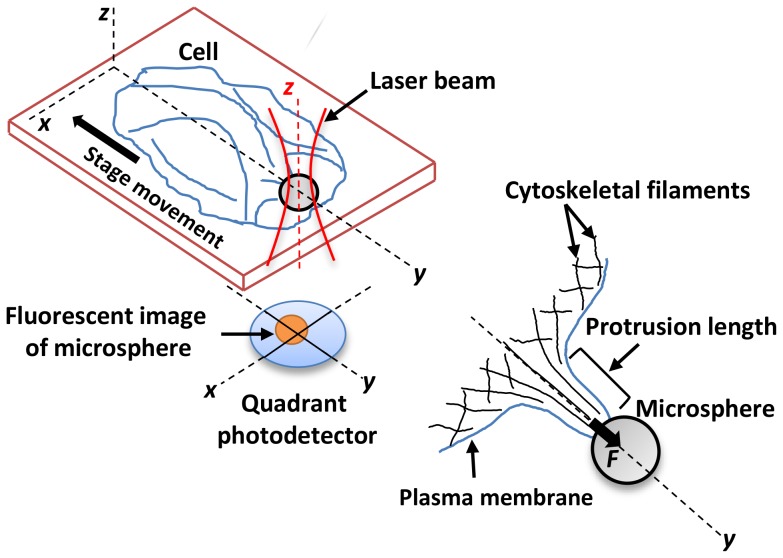
Schematic of a protrusion induced on an adherent living cell using optical tweezers. The protrusion forms in response to application of a tensile force (*F*) on the cell surface in *y* direction by a laser trapped microsphere (bead) (drawn not to scale). A quadrant photodetector records the instantaneous displacement of the trapped microsphere from the trapping center for force measurements while the stage is driven in the negative *y* direction.

We optically trapped 4 µm diameter (*d*) sulfate-modified fluorescent polystyrene microspheres (beads) (F-8858, Molecular probes, Eugene, OR), and used them as handles to induce local cell deformation, in the form of a protrusion, by pulling the cell away from the bead (Fig. 1). An infrared Nd:YVO_4_ diode-pumped solid state laser (1064 nm, Prisma-1064-8-V, Coherent) was used to create the optical tweezers [13]. The optical tweezers setup consisted of an inverted microscope (Nikon Eclipse Ti-DH) containing a 100X oil immersion objective with high numerical aperture (NA = 1.49) (Nikon, Apo TIRF) through which the laser beam was passed and converged to form an optical trap. The laser beam was expanded prior to entering the microscope to fill the back aperture of the microscope objective.

The excitation spectrum of the fluorescent beads is between 480 and 590 nm with maximum fluorescence emission at 605 nm. Excitation light from a mercury source (Nikon, Intenslight, C-HGFI) passed through a filter set (Nikon, TRITC, TE 2000) to illuminate the trapped bead. The filter set included an excitation filter (525–560 nm), a dichroic mirror that reflected the excitation light into the objective to illuminate the trapped fluorescent bead, and an emission filter (570–620 nm), which transmitted the emitted light from the bead. An emission bandpass filter (Chroma, 605±25 nm) was placed in front of a position sensing quadrant photo-detector (QPD) (QP50-6SD, Pacific silicon sensor, Westlake village, CA) to specifically select the fluorescent emission from the bead.

We optically monitored the displacement of the bead as a method to determine forces associated with protrusion formation and elongation. The fluorescent image of the trapped bead was projected onto the center of the QPD to measure the displacement of the trapped bead from the trapping center. Using an analog-to-digital converter (Wavebook 521, IOTEch, Cleveland, OH), the sum-and-difference output signals (in mV) from the QPD amplifier were digitized and subsequently recorded by WaveView software (WaveView 7.14.16, IOTech). The collection frequency was 66.66 Hz. A charge-coupled device (CCD) camera (Hamamatsu Corp., EM-CCD, C9100/13, Bridgewater, NJ) was used to visualize the objects in the field of view.

The change in QPD output voltage signal is proportional to an external force such as the force exerted by the cell onto the bead. The output voltage signal of the QPD was calibrated for the external force using the viscous drag force method based on Stokes' Law [20]. In this method, a known drag force was applied to the trapped bead while recording the resulting differential signal from the QPD. The drag force was generated by driving a piezoelectric translation stage (PZT) (Physik Instrumente, Model P-527.C3, Waldbronn, Germany) at known velocities. The resolution of the piezoelectric stage was 10 nm in *x* and *y* directions, and 2 nm in *z* directions (laser beam propagation direction). The applied force was linearly fit to the output voltage of the QPD (*R* ^2^ = 0.99). In this study, calibration and force measurements were performed at laser power of 350 mW after the microscope objective with trap stiffness typically ranging between ≈100 and 200 pN/µm. There was no evidence of thermally induced structural damages in the living cells at this power and trapping wavelength. The relationship between the bead displacement and QPD signal was determined by applying known displacements to a coverslip-immobilized bead while recording the QPD voltage signal. There was a linear relationship between the bead displacement and QPD output signal (*R* ^2^ = 0.97). We measured both voltage-force and voltage-displacement relationships before each experiment.

### Protocol for formation of cell protrusion

Once a bead was optically trapped, a cell firmly attached to the bottom of the Petri dish, was brought to proximity (≈0.5 µm) of the bead using the PZT stage. We then triggered the PZT to move the cell toward the trapped bead at 10 nm steps until they were in contact, as determined by appearance of a non-zero differential QPD signal. The mechanism of the bead-cell membrane attachment in our experiments is non-specific, and involves electrostatic interactions between the sulfate-modified polystyrene beads and the cell plasma membrane.

The bead and the cell were in contact for ≈5–10 seconds to achieve plasma membrane-bead attachment, and then the cell was subsequently moved away from the bead at 1 µm/s, forming a protrusion (Fig. 1). A sequence of images showing the protrusion formation process in our experiments is provided in the Supporting Information (Fig. S1 and Text S1). The movement of the cell continued to the point where the plasma membrane separated from the cytoskeleton as determined by a sudden reduction in force from its maximum value. An illustrative temporal force profile is presented in the Supporting Information (Fig. S2 and Text S2).

Fluorescence imaging of the trapped microsphere eliminated the force measurement artifact that arises under bright-field illumination when the microsphere is in proximity of the cell [21], [22]. Assuming the indentation depth (Δ*h*) produced by a 4 µm diameter microsphere in the plasma membrane to be ≤10 nm, the patch radius is estimated as *r*_p_≤*d*Δ*h* or *r*_p_≤0.2 µm [21]. Therefore, with patch area defined as *A*_p_ *= πr*_p_^2^, it was estimated as *A*_p_≤0.126 µm^2^.

### Measurement of cell protrusion length

Length of the cell protrusion (*x*_pt_) was determined by using the instantaneous piezoelectric stage displacement (*x*_PZT_), and the transverse displacement of the trapped bead from the trapping center (*x*_bead_) in the pulling direction (Eq. 1) [21]:
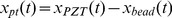
(1)


**Figure 2:**
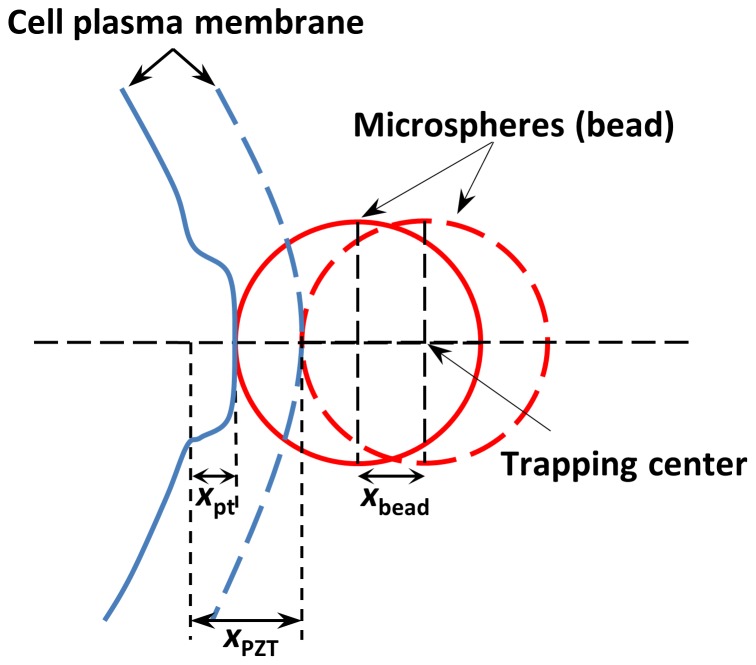
Schematic of bead-cell contact system. Dashed lines show initial cell-bead contact where there is no external force applied on the bead. Solid lines show the bead-cell system once the cell is moved away from the trapped bead by distance *x*_pzt_. Bead is displaced from the trapping center by *x*_bead_ and the length of the protrusion is *x*_pt_.

The schematic of a bead-cell system is shown in Fig. 2 wherein the cell and the bead are initially in physical contact prior to formation of the protrusion. This stage is shown by dashed lines on the figure, and is followed by a subsequent stage where a protrusion of length *x*_pt_ is formed by pulling the cell away from the bead by moving the PZT stage over a distance *x*_PZT_. This stage is shown by solid lines where the bead is displaced by *x*_bead_ from the trapping center.

### Physical model

**Figure 3:**
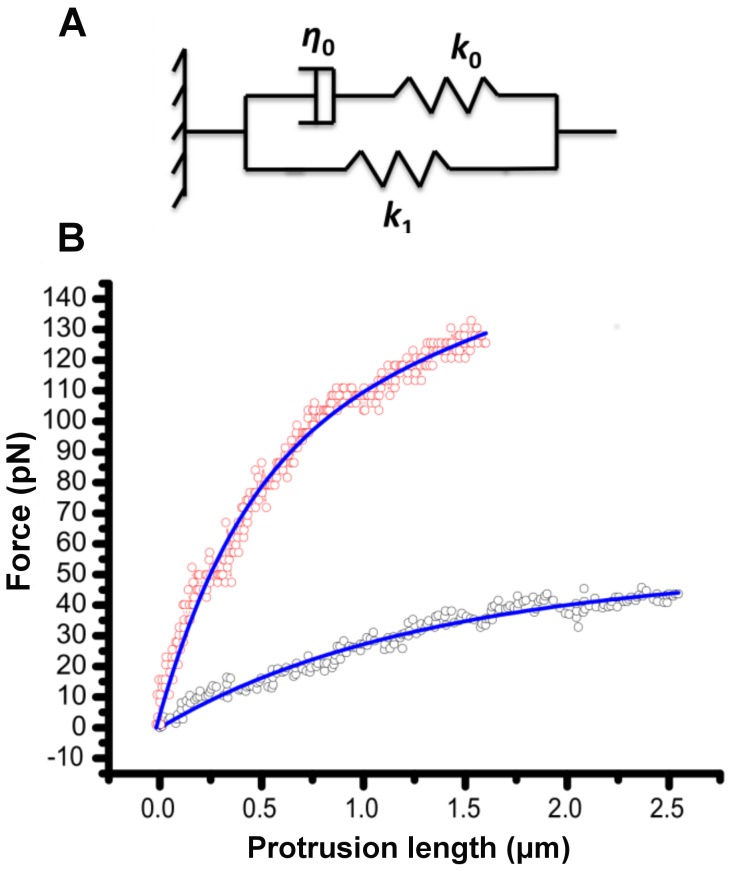
Cell protrusions as a viscoelastic structure. (**A**) Standard linear solid (SLS) model of the protrusion. The model includes a Maxwell arm (viscous coefficient of *η_0_* in series with spring with stiffness *k*_0_) in parallel with a spring with stiffness *k*_1_. (**B**) Two illustrative force-length profiles of protrusion elongation fitted with the SLS model (blue solid lines). The red circles show data for a cell with intact F-actin and normal membrane cholesterol content. Data in black circles are from cells with disrupted F-actin using Latrunculin-A, but with and normal membrane cholesterol level.

The instantaneous force-length plots (*F*-*x*_pt_) were obtained from optical tweezers experiments under constant retraction velocity. We examined three standard viscoelastic models: Voigt, Maxwell, and Standard Linear Solid (SLS) (Kelvin) model to fit the measured force profiles of the cell protrusions [23]–[25]. The SLS model was able to fit both the protrusion force-length (*F*-*x*_pt_), and force relaxation profiles under constant protrusion length in our experiments. The SLS model consists of a Maxwell body in parallel with a spring of stiffness *k*_1_, where the Maxwell body itself includes a spring with stiffness *k_0_* connected in series with a dashpot having viscous coefficient of *η_0_* (Fig. 3A). The governing equation of the SLS model applied to the cell protrusion is [24], [25]:
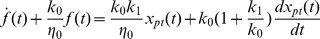
(2)where *f*(t) and *x*_pt_(t) are the instantaneous protrusion force, and protrusion length, respectively. The rate of change in protrusion length (*V* = d*x*_pt_/dt) was measured as the slope of the *x*_pt_-time plots. With *t* = *x_pt_*/*V* and *f*(*x_pt_* = 0) = *F*_0_, Eq. 2 can be converted to Eq. 3 as:
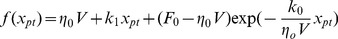
(3)


We present illustrative force-length profiles for an intact F-actin and an F-actin disrupted cell fitted with the SLS model (Fig. 3B). Force values increase with protrusion length, and reach a maximum value (*F*_max_) where the plasma membrane becomes separated from the underlying cytoskeleton indicated by a sudden drop in the force value (Fig. S2). We refer to the protrusion length associated with this maximum force value as maximum protrusion length (*l*_pt-max_). We fit Eq. 3 to the measured force-length profiles to extract the values of *η*_0_, *k*_0_, and *k*_1_ associated with the SLS model. We excluded from our analyses the cases where there was a rupture event during the protrusion formation experiments as evidenced by an abrupt force drop in the force-length profiles.

### Protocol for reverse pull experiments

**Figure 4:**
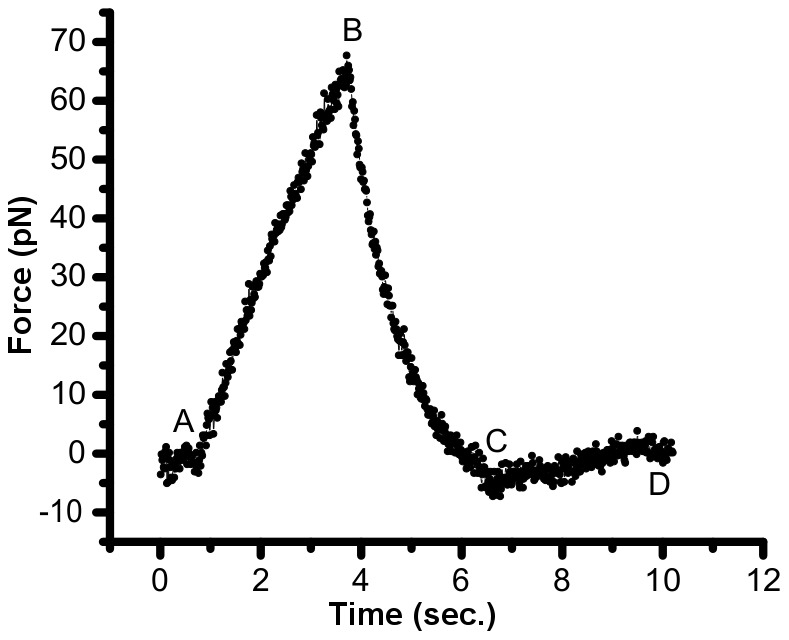
Reverse pull experiment. An example force-time plot involving subsequent “pull” and “push” processes. The ∼1 second time interval between zero second and point A indicates bead-cell contact prior to the push-pull experiments beginning at A.

The cell was moved away from the trapped bead at 1 µm/s for 3 µm (pull process) and immediately returned at the same velocity to the starting point (push process) under this protocol. A force-time plot of an example reverse pull experiment is shown in Fig. 4, representing two different segments: 1- pull (AB segment) where the cell is moved away by 3 µm at 1 µm/sec, and 2- push (BC segment) where the cell is moved back by 3 µm at 1 µm/sec. At the end of the push process at state C, force value is slightly smaller than that of before pull process at state A. This slight reduction in force value indicates that the bead passed its original position towards the negative direction. The cell restores its shape as the protrusion disappears within several seconds, and the force level reaches state D where the value of the force was nearly the same as that at the beginning of the pull process at state A. The force-time profiles of the reverse pulling experiments were converted to force-length profiles based on the method described earlier in the Materials and Methods in Eq. 1. Under this protocol, we quantified the percent of energy loss (*W*_loss_) in terms of (*W*_pull_-*W*_push_)/*W*_pull_, where *W*_pull_ and *W*_push_ are energies associated with pulling and pushing the protrusions determined as the areas under the force-length curves. The cases where membrane-cytoskeleton separation occurred during the pulling process were not considered in our analysis of the reverse pull protocols.

### Statistical analysis

We used a standard two-sample student's t-test with unknown variances of two data sets in our statistical analysis. Statistical significance was accepted if the *P*-value was <0.05.

## Results

### Maximum protrusion force (*F*_max_)

**Figure 5:**
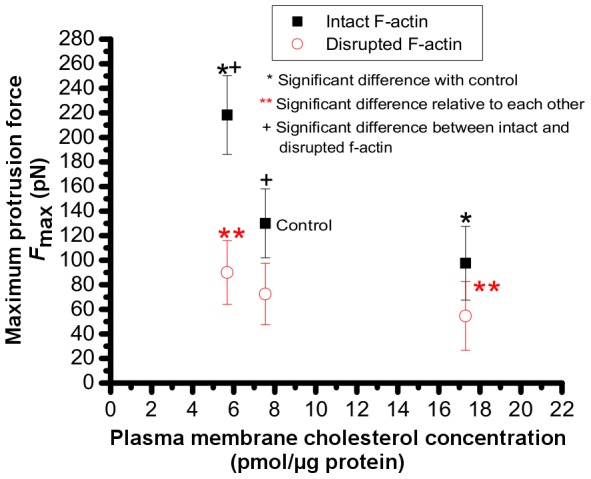
Effect of membrane cholesterol content and cytoskeletal F-actin on maximum protrusion force. Maximum protrusion force (*F*_max_) is defined as the force that results in separation of membrane from cytoskeleton.

The maximum protrusion force (*F*_max_) is the force at which the plasma membrane separates from the cytoskeleton. The values of *F*_max_ for cells with intact and disrupted F-actin as a function of membrane cholesterol content are shown in Fig. 5. Cholesterol depletion resulted in a statistically significant increase in the values of *F*_max_, whereas cholesterol enrichment decreased it. Specifically, mean±standard deviation (s.d.) values of *F*_max_ for control HEK cells (cells with intact F-actin network and normal cholesterol content) was 130±28 pN (n = 16), and significantly increased to 218±32 pN (n = 7) when cell were incubated in DMEM containing 5 mM MβCD for cholesterol depletion. In response to incubation of the cells in DMEM containing 5 mM cholesterol-MβCD for cholesterol enrichment, the mean±s.d. values of *F*_max_ significantly decreased to 96.72±29 (n = 13) (*P*<0.05).

Disruption of F-actin by treatment of the cells with Latrunculin-A resulted in a statistically significant reduction in *F*_max_ values from 130±40 to 72.44±25 pN in control cells whose cholesterol contents were not manipulated. Values of *F*_max_ also decreased in cholesterol manipulated cells upon F-actin disruption; specifically from 218±45 to 90±26 pN (*P*<0.05) in cholesterol depleted cells, and from 96.72±29 to 55±28 pN (*P*<0.05) in cholesterol enriched cells. Comparing the forces among the cells with disrupted F-actin, we found no statistically significant difference between control and cholesterol enriched cells, nor between control and cholesterol depleted cells.

### Maximum protrusion length (*l*_pt-max_)

**Figure 6:**
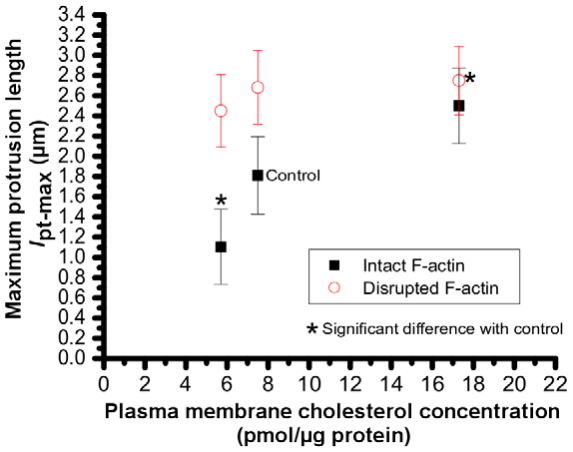
Effect of membrane cholesterol content and cytoskeletal F-actin on maximum protrusion length. Maximum length of protrusion (*l*_pt-max_) is defined the length at which the membrane becomes separated from the cytoskeleton.

The maximum protrusion length (*l*_pt-max_) is the length associated with *F*_max_ at which the plasma membrane becomes separated from the underlying cytoskeleton. For control cells, this length was 1.81±0.38 µm. It decreased upon cholesterol depletion to 1.1±0.37 µm, and increased to 2.5±0.37 µm in response to cholesterol enrichment (Fig. 6). Disruption of F-actin increased the *l*_pt-max_ to 2.68±0.36 µm in cells with normal cholesterol content, and to 2.45±0.36 in cholesterol depleted cells. The value of *l*_pt-max_ in cholesterol enriched cells after disruption of F-actin was 2.75±0.34 µm.

### Stiffness and coefficient of viscosity

**Figure 7:**
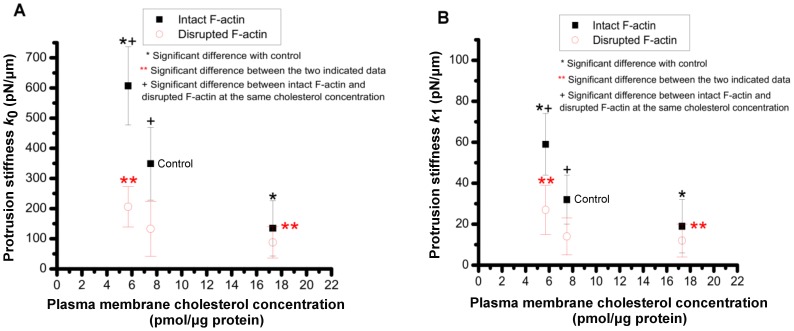
Protrusion stiffness. Effect of membrane cholesterol content on: (**A**) stiffness parameter *k*_0_, and (**B**) stiffness parameter *k*_1_ for cells with intact or disrupted F-actin.

The stiffness parameters (*k*_0_, and *k*_1_) and coefficient of viscosity (*η*_0_) were obtained from the SLS model applied to protrusion force-length plots. The mean±s.d. value of *k*_0_ for control HEK cells was estimated as ≈349±121 pN/µm (Fig. 7A). There was a statistically significant increase in *k*_0_ to ≈607±130 pN/µm for cholesterol-depleted cells, and a decrease to ≈135±92 pN/µm for cells under cholesterol enrichment condition. Disruption of F-actin resulted in significant decrease in the values of the stiffness for normal and cholesterol depleted cells to ≈133±91 and 206±67 pN/µm, respectively (*P*<0.05). The stiffness of the cholesterol enriched cells was not significantly changed upon F-actin disruption (≈88±52 pN/µm).

The mean±s.d. value of *k*_1_ for control cells was ≈32±15 pN/µm. It significantly increased to ≈59±15 pN/µm in response to cholesterol depletion, and decreased significantly to ≈19±13 pN/µm for cells under cholesterol enrichment condition (Fig. 7B). Disruption of F-actin was associated with significantly decreased values of *k*_1_ in control cells to ≈14±9 pN/µm and to ≈27±12 pN/µm in cholesterol depleted cells. The values of *k*_1_ for cholesterol enriched cells after F-actin disruption was ≈12±8 pN/µm.

**Figure 8:**
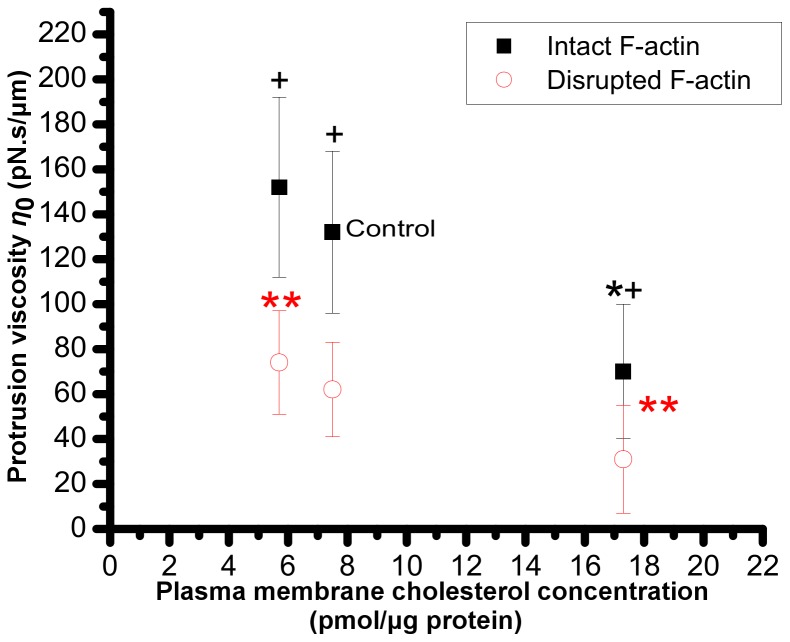
Protrusion viscosity. Effect of membrane cholesterol content on the viscosity coefficient of the protrusion for cells with intact or disrupted F-actin. Asterisk (*) indicates a statistically significant difference with control; ** indicates a statistically significant difference between the two indicated data sets; and + indicates a statistically significant difference between intact F-actin and F-actin disrupted cells at the same cholesterol concentration.

The values of viscosity coefficient (*η*_0_) were ≈132±36 and 71±30 pN.s/µm for control and cholesterol-enriched cells, respectively (Fig. 8), and did not change significantly in cholesterol-depleted cells with respect to control cells. In comparison to cells with intact F-actin, disruption of F-actin significantly decreased *η_0_* to 62±21, 31±24, and 74±23 pN.s/µm in cells with normal cholesterol level, cholesterol-enriched, and cholesterol-depleted cells, respectively.

## Discussion

### Plasma membrane cholesterol level modulates cellular protrusions

Cholesterol depletion increased the values of *F*_max_ and stiffness values of *k*_0_, *k*_1_ associated with protrusions, and resulted in shorter protrusions, whereas cholesterol enrichment decreased the value of *F*_max_, *k*_0_, *k*_1_ and resulted in longer protrusions. Effects of cholesterol on protrusion stiffness at different times during the protrusion formation process can be analyzed through two stiffness parameters associated with the SLS model. In principle, based on the SLS model, the early stage of the protrusion formation is controlled by *k*_0_+*k*_1_ as the instantaneous stiffness modulus, while towards the end of the protrusion formation process, the parameter *k*_1_ becomes dominant as the long-term stiffness modulus. Based on our results, the stiffness parameter *k*_0_ is significantly greater than *k*_1_ under all conditions examined; therefore, the instantaneous stiffness modulus (*k*_0_+*k*_1_) at the early stage of protrusion formation is dominated by *k*_0_ (Figs. S3–S5 and Text S3 in Supporting Information for parametric analysis).

We recently probed the mechanical properties of HEK cell plasma membranes by pulling membrane tethers under membrane cholesterol levels identical to the ones examined in this study for protrusions [13]. In tether pulling experiments, membrane stiffness and viscosity in cells with intact F-actin and normal cholesterol content (control cells) were estimated as ≈2 pN/µm and 2.6±1 pN.s/µm, respectively. In this study, the mean±s.d values of protrusion viscosity (*η*_0_) for control (without cholesterol manipulation) cells with intact F-actin are ≈132±36 pN.s/µm, and the values of protrusion stiffness *k*_0_ and *k*_1_ are ≈349±121 and ≈32±15 pN/µm, respectively. Given that the values of stiffness and viscosity of cellular protrusions are significantly greater than those for membrane tethers, the plasma membrane effects are relatively minor in protrusions. The minor effects of membrane mechanical properties will be more evident by comparing them with those associated with protrusions formed from F-actin disrupted cells. Membrane tether viscosity and stiffness still remained noticeably lower than the values of *η*_0_ (62±21 pN.s/µm), *k*_0_ (133±91 pN/µm), and *k*_1_ (14±9 pN/µm) in protrusions from F-actin disrupted cells without cholesterol manipulation. Additionally, disruption of F-actin did not result in a significant change in the viscous coefficient of the tethers for the same levels of membrane cholesterol content [13]. However, F-actin disruption significantly reduced the viscous coefficient of the protrusions. The stiffness and viscosity values of membrane tethers and surface protrusions are summarized in Table 1 for cells with intact and disrupted F-actin under different membrane cholesterol levels.

**Table 1:** Plasma membrane and protrusion stiffness and viscosity.

Plasma membrane cholesterol content (pmol/µg protein)	Membrane tether stiffness (pN/µm) [13]	Membrane tether effective viscosity (pN.s/µm) [13]	Protrusion stiffness (*k*_0_) (pN/µm)	Protrusion stiffness (*k*_1_) (pN/µm)	Protrusion viscosity (*η*_0_)(pN.s/µm)	Protrusion stiffness (*k*_0_) + Lat-A (pN/µm)	Protrusion stiffness (*k*_1_) + Lat-A (pN/µm)	Protrusion viscosity (*η*_0_)+ Lat-A (pN.s/µm)
5.68	3.24±0.1	Not determined¶	607±130	59±15	152±40	206±67	27±12	74±23
7.54	1.2±0.19	2.6±1	349±121	32±15	132±36	133±91	14±9	62±21
17.3	0.28±0.01	3.6±0.73	135±92	19±13	71±30	88±52	12±8	31±24

Plasma membrane cholesterol concentration in control cells is 7.54 pmol/µg protein. Lat-A represents treatment of the cells with Latrunculin-A in order to disrupt F-actin polymerization.

¶ Membrane tether effective viscosity was not determined at 5.68 pmol/µg protein cholesterol concentration, but its value under cholesterol depleted conditions at 6.6±0.3 pmol/µg protein was ≈2.1±0.4 pN.s/µm [13].

Given the relatively major contribution of cytoskeleton, the observed variations in the values of *F*_max_, *k*_0_, and *k*_1_ in cholesterol-manipulated cells reflect the corresponding effects of cholesterol on plasma membrane-cytoskeleton connections and the underlying cytoskeleton within the protrusions. The respective tether stiffness values of 3.24±0.1 and 0.28±0.01 pN/µm from cholesterol depleted and cholesterol enriched cells, are orders of magnitude smaller that the corresponding *k*_0_ protrusion stiffness values of 607±130 pN/µm and135±92 pN/µm (Table 1), indicating the relatively major effects of the cytoskeleton at the early stage of protrusion formation. Comparing the values of tether stiffness with the *k*_1_ values of 59±15 pN/µm for cholesterol depleted and 19±13 pN/µm for cholesterol enriched cells, the effects of cytoskeleton continue to remain major at the end stage of protrusion formation process as well.

By forming plasma membrane tethers in a recent study [13], we quantified the plasma membrane-cytoskeleton adhesion energy per unit area in cells with intact and disrupted F-actin under various cholesterol levels identical to those in this study. For example, in comparison to control cells with normal cholesterol content, we found that the membrane-cytoskeleton adhesion energy in cholesterol depleted cells increased by nearly four-fold. Interestingly, when the cytoskeletal F-actin was disrupted with Latrunculin-A following cholesterol manipulation, nearly the same value of in-plane tension (≈5×10^−18^ J/µm^2^) within the membrane was obtained regardless of the plasma membrane cholesterol level. This observation indicates that changes in the plasma membrane-cytoskeleton adhesion energy were in response to cholesterol treatments, and not due to the bead-membrane adhesion.

Taken together, our observations indicate the effects of cholesterol at both the early and late stages of protrusion formation within the cholesterol concentration range examined. These observations are in accordance with the previous observations in cholesterol-depleted bovine aortic endothelial cells where greater negative pressures were required to locally deform and aspirate part of the cells in micropipette aspiration measurements [26], [27]. Those investigators suggested that the stiffening induced by cholesterol depletion could be either due to effects on the submembrane cytoskeleton F-actin, or actin-membrane connections. In another study, neutrophils with elevated membrane cholesterol content exhibited increased whole cell deformability, suggesting the effects of cholesterol enrichment on submembrane structures [28]. It is worth noting that the lower values of *k*_1_ compared to *k*_0_ could be attributed to the shape change processes underlying protrusion formation and elongation. The *k*_0_ stiffness, which is dominant at the early stage of protrusion formation, and has a higher value than *k*_1_, corresponds to a conical-shape protrusion. At the late stage of protrusion elongation with subsequent initiation of tubular (tether) formation, the *k*_1_ stiffness whose value is smaller than *k*_0_ and closer to the tether stiffness, becomes dominant.

The effects of cholesterol on non-biological membranes show dependency on the number of saturated hydrocarbon chains in the lipids [29]–[31]. Specifically, membrane bending modulus increases with added cholesterol in lipids with two saturated chains such as 1,2-dimyristoyl-snglycero-3-phosphocholine lipids [32]. However, when both chains are monounsaturated as in 1,2-dioleoyl-sn-glycero-3-phosphocholine and 1,2-dierucoyl-sn-glycerol-3-phosphocholine lipids, the bending modulus remain unchanged for cholesterol mole fractions up to 0.4 [32]. In giant bilayer vesicles, the bilayer cohesion increases with added cholesterol only for lipids in which both chains are saturated or monosaturated. Specifically, the area expansion modulus of stearoyl-oleoyl phosphatidylcholine type of lipids is reported to increase by six times with addition of cholesterol up to 58 mol% [33]. Lipid vesicles lack the cytoskeletal effects which are present in living cells. Therefore, effects of cholesterol manipulations on cytoskeletal structures and plasma membrane-cytoskeleton adhesion would be absent in experiments performed on vesicles. The actin network in cytoskeleton also controls the spatial and temporal organization of the membrane domains. Actin polymerization can induce and actively organize the formation of membrane domains through cytoskeleton-liked lipid-protein interaction, while the disassembly of actin network can abolish the organization of the membrane [34].

While according to our results, the mechanical properties of the protrusions are dominated by the cytoskeleton, membrane properties are important in protrusion formation processes. Depletion of human colon adenocarcinoma cells with cholesterol by MβCD inhibited the initiation of microtubule-based protrusion formations dose-dependently in cells treated with clostridium difficile toxin [11]. The authors of that study suggested that inhibition of protrusion initiation is likely attributed to changes in biophysical properties of the membrane upon cholesterol depletion rather than changes in microtubule dynamics. The higher stiffness values of the membrane under cholesterol depletion [13] can be a potential contributor to this observation. In another study, high membrane cholesterol concentrations reduced shear response of the polymorphonuclear leukocytes with attenuating effects on pseudopodia formation, suggesting a correlation between plasma membrane fluidity and cell mechanotransduction [12].

The effects of cytoskeleton on protrusions can be investigated by disruption of the F-actin network. Latrunculin-A binds to monomeric G-actin to inhibit F-actin assembly without impacting cell viability or interfering with microtubules and intermediate filaments [17], [18], [35], [36]. Therefore, the observed changes in the values of *F*_max_, *l*_pt-max_, *k*_0_, *k*_1_, and *η_0_* in Latrunculin-A treated cells reflect the effects of F-actin on protrusions. Cholesterol enrichment/depletion resulted in opposing effects, while disruption of F-actin diminished the disparity between the cholesterol-manipulated cells and the cells with normal cholesterol content. These observations are interpretable in terms of the effects of cholesterol on actin-membrane connections, and cytoskeleton components within the protrusions, which are decreased in the absence of F-actin network.

### Viscoelastic behavior of cellular protrusions

The force-length plots associated with protrusions hand non-linear profiles (Fig. 3). This observation is attributed to breakage of the bonds between plasma membrane and cytoskeleton with subsequent flow of membrane lipids into the protrusion, and additional viscous effects due to the slip between the plasma membrane and cytoplasm during protrusion formation [37]–[40]. The presence of viscous effects and bond breakage manifest themselves as hysteresis in our reverse pull experiments.

**Figure 9:**
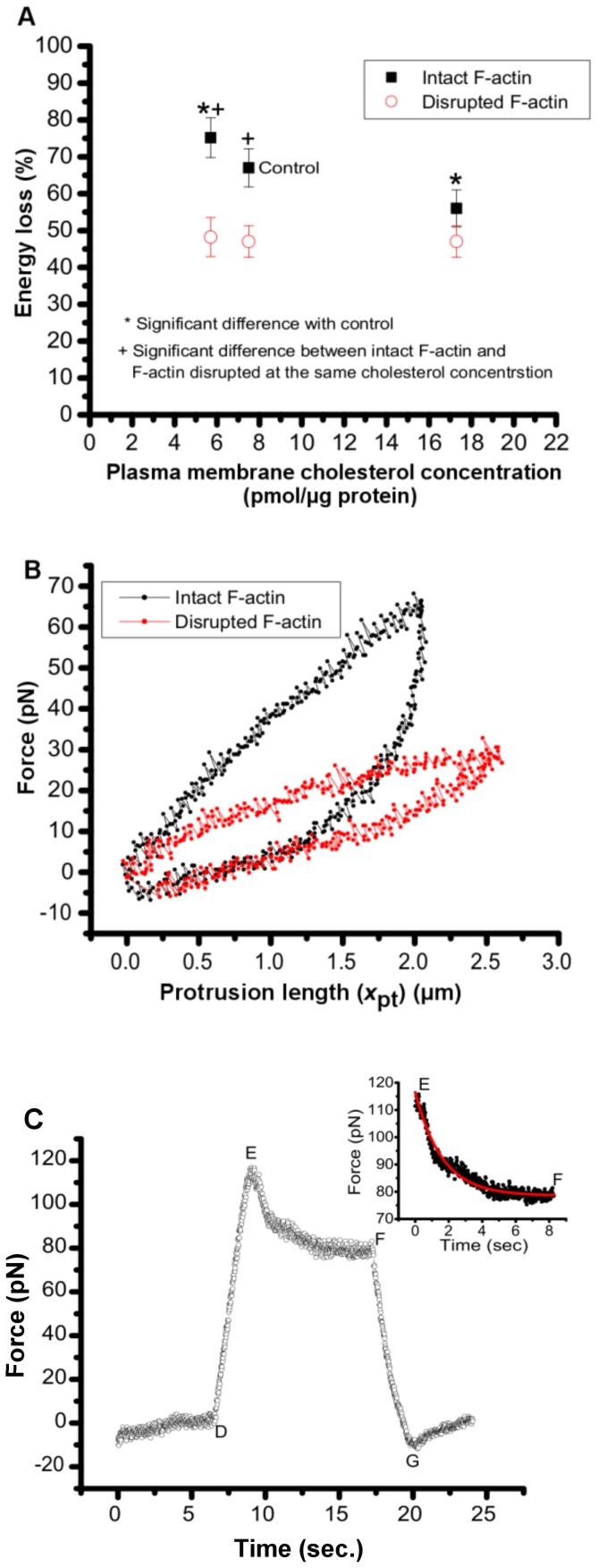
Energy loss associated with protrusion hysteresis. (**A**) Effect of membrane cholesterol content on the percent of energy loss associated with protrusion hysteresis for intact and disrupted F-actin cells. (**B**) Force-length plots indicating hysteresis in cells with intact and disrupted F-actin. lpar;**C**) Protrusion force during protrusion elongation to 3 µm (time interval D–E) followed by force relaxation after the protrusion reaches 3 µm, and maintained at that length (time interval E–F). The interval F–G displays the force as the protrusion is unloaded (pushed back). The inset shows the SLS-fit to the force relaxation during the time interval between the end of the pulling time and beginning of the pushing time (E–F). The general solution for the protrusion force relaxation under constant length is: 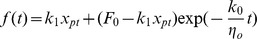, where *x*_pt_ is the constant length of the protrusion (3 µm), and *F*_0_ is the force at the beginning of relaxation. The ∼7 seconds time interval between zero second and point D indicates bead-cell contact prior to the push-pull experiments beginning at D.

The *W*_loss_ in control cells is 67±6%, close to the value of 53±15% reported for human neutrophils [41] (Fig. 9A). Our analysis indicate the cytoskeleton dependency of energy loss in protrusion formations, as evidenced by the contraction of the hysteresis loop (Fig. 9B), and the significant decrease of *W*_loss_ to 47±4% in control cells after disruption of F-actin. The more energy efficient protrusion formation in Latranculin-A treated cells may be the result of fewer cytoskeleton-membrane connections coupled with the observed decrease in protrusion viscosity.

The SLS model fits the force-length plots in cells with intact F-actin and F-actin disrupted cells (Fig. 3), indicating the viscoelastic behavior of the protrusions. To further investigate the viscoelasticity of the protrusions, we investigated their force relaxation. To this end, in a reverse-pull experiment, protrusion pulling was stopped at the end of the pulling process, resulting in immediate relaxation of protrusion force in a cell with intact F-actin (Fig. 9C). The force relaxation continued until reaching an equilibrium value prior to the push-back process. The presence of both force relaxation and hysteresis effects in these protrusions indicates the viscoelastic behavior of the protrusions [41]. The force relaxation plot was fitted with the SLS model (Fig. 9C inset).

The observed decreased values of protrusion viscosity upon F-actin disruption are consistent with the previous observations showing the dose-dependent decreasing effects of F-actin disruption on cytoplasmic viscosity [42], [43]. The less viscous and more fluid behavior of the cytoplasm in F-actin disrupted cells can be attributed to the reduced opposing effects of cytoskeleton microfilaments on cytoplasm movement. Therefore, the formation of longer protrusions prior to membrane-cytoskeleton separation in F-actin disrupted cells and cholesterol enriched cells could be due to their faster growth during protrusion formation as a result of their lower values of viscosity. This observation is consistent with the SLS model predictions (Fig. S3 and Text S3) where protrusions with lower values of viscosity coefficient are associated with higher lengths under the same pulling force. Similarly, protrusions with lower stiffness values of *k*_0_, and *k*_1_, as in the cases of F-actin disrupted cells and cholesterol enriched cells, are associated with longer protrusions under the same force (Figs. S4, S5 and Text S3). Greater protrusion stiffness values either at the early stage of protrusion formation (dominated by *k*_0_) or towards the end of the protrusion elongation (dominated by *k*_1_), such as those induced by cholesterol depletion, are predicted to result in shorter protrusions by the SLS model. Previous experiments performed on microvascular endothelial cells [44] and terminally differential fibroblasts [45] resulted in formation of longer tethers in response to disruption of F-actin; specifically, length of tethers pulled from fibroblasts increased four-fold as a result of F-actin disruption [45].

The force-length plots associated with neutrophil surface protrusions have been modeled as a Voigt element in series with a linear spring [34], [39]. The SLS model used in this study describes membrane-cytoskeleton behavior in HEK cells during protrusion formation and force relaxation under constant length, with a viscous coefficient and two stiffness parameters. Each stiffness parameter is dominant at the early or end stage of protrusion formation. The SLS model can be extended to interpret some other biological processes. For example, a generalized form of the SLS model, wherein a Maxwell body was placed in parallel to the SLS system, has been used to interpret the fast and slow dynamic responses involved in biphasic force relaxation in plasma membrane tethers pulled from outer hair cells, sensory cells within the cochlea in auditory system [46]. We recognize that other mechanical models may be appropriate to capture microrheological behavior involved in cellular deformations over extended time scales and frequency ranges of deformation [47]–[50].

Taken together, plasma membrane and cytoskeleton mutually contribute to the viscoelastic behavior of the cellular protrusions. Effects of plasma membrane mechanical properties are relatively minor in comparison with cytoskeleton effects; however, surprisingly modulation of membrane composition resulted in significant changes in protrusion mechanics. Membrane cholesterol depletion and enrichment showed opposing effects on various mechanical properties of protrusion while disruption of F-actin diminished the observed disparities. These observations suggest a correlation between membrane and protrusion mechanical properties through modifications in plasma membrane-cytoskeleton connections and underlying cytoskeleton properties in response to changes in membrane mechanical properties. The results of this study provide insight into understanding the membrane-induced modulations of cellular protrusions and mechanotrasduction.

## Supporting Information

Figure S1 **Sequence of images showing the formation of a cellular protrusion.** An adherent cell was brought in contact with an optically trapped bead. After initial contact was established between the cell and the bead, the cell was moved away using a piezoelectric stage, resulting in formation of a local deformation (protrusion) at the cell surface (images 1–10). A transition from cellular protrusion to a tether formation occurs (image 11) in response to further elongation of the protrusion resulting in formation of a tether (images 12–15). The images were taken by optical microscopy under white-light illumination regime with a 100X objective lens (NA = 1.49). The time interval for this sequence of images was ≈6 seconds (≈400 ms between images). Scale bars = 5 µm. Data for this study were obtained prior to tether formation (images 1–10). This sequence of images was obtained from one membrane pulling experiment (see Text S1).
(TIF)

Figure S2 **Force trajectory associated with application of a tensile force over cell surface.** The pulling process starts with a rise in the force (AB) till reaching a first peak at B, followed by a drop in the force value from B to C. The force value reaches a second peak (D) at the end of pulling process. The force relaxation process starts once the pulling is halted. Pulling velocity is 1 µm/sec, and pulling length is 20 µm over the time interval A–D. This tether force profile is representative, and resembles other such profiles as reported in our previous publications (see Text S2).
(TIF)

Figure S3 **Effects of changes in** ***η_0_*** **on protrusion force-length plots based on SLS model.** Values of parameters *k*_0_ and *k*_1_ are kept constant at 350 pN/µm and 30 pN/µm, respectively, while *η_0_* (pN.s/µm) changes as shown. The SLS model predicts formation of longer protrusion associated with lower values of viscosity in response to a given force value. Effects of the viscous parameter become more pronounced at the later stages of protrusion formation.
(TIF)

Figure S4 **Effects of changes in** ***k*** **_0_ on protrusion force-length plots based on SLS model.** Values of parameters *k*_1_ and *η_0_* are kept constant at 30 pN/µm and 100 pN.s/µm, respectively, while *k*_0_ (pN) changes as shown. The profiles indicate the effects of the *k*_0_ stiffness at the early stage of the protrusion formation, and formation of shorter protrusion under a given force value in response to higher values of *k*_0_ stiffness.
(TIF)

Figure S5 **Effects of changes in** ***k*** **_1_ on protrusion force-length plots based on SLS model.** Values of parameters *k*_0_ and *η_0_* are kept constant at 350 pN/µm and 100 pN.s/µm, respectively while *k*_1_ (pN) changes as shown. The *k*_1_ stiffness affects the late stage of the protrusion formation. Higher values of *k*_1_ are associated with formation of shorter protrusions under a given force value.
(TIF)

Text S1 **Imaging the cell protrusion formation.**
(DOC)

Text S2 **Illustrative dynamic force profile.**
(DOC)

Text S3 **Parametric analysis.**
(DOC)

## References

[1] Allena R, Aubry D (2012) ‘Run-and-tumble’ or ‘look-and-run’? A mechanical model to explore the behavior of a migrating amoeboid cell. J Theor Biol 306: 15–31. 22726805 10.1016/j.jtbi.2012.03.041

[2] Bergert M, Chandradoss SD, Desai RA, Paluch E (2012) Cell mechanics control rapid transitions between blebs and lamellipodia during migration. Proc Natl Acad Sci USA 109: 14434–14439. 22786929 10.1073/pnas.1207968109 PMC3437886

[3] Tatavarty V, Das S, Yu J (2012) Polarization of actin cytoskeleton is reduced in dendritic protrusions during early spine development in hippocampal neuron. Mol Biol Cell 23: 3167–3177. 22740628 10.1091/mbc.E12-02-0165 PMC3418311

[4] Evans E, Heinrich V, Leung A, Kinoshita K (2005) Nano- to Microscale Dynamics of P-Selectin Detachment from Leukocyte Interfaces. I. Membrane Separation from the Cytoskeleton. Biophys J 88: 2288–2298. 15653718 10.1529/biophysj.104.051698 PMC1305278

[5] Heinrich V, Leung A, Evans E (2005) Nano- to Microscale Dynamics of P-Selectin Detachment from Leukocyte Interfaces. II. Tether Flow Terminated by P-Selectin Dissociation from PSGL-1. Biophys J 88: 2299–2308. 15653735 10.1529/biophysj.104.051706 PMC1305279

[6] Ramachandran V, Williams M, Yago T, Schmidtke DW, McEver RP (2004) Dynamic alterations of membrane tethers stabilize leukocyte rolling on P-selectin. Proc Natl Acad Sci USA 101: 13519–13524. 15353601 10.1073/pnas.0403608101 PMC518789

[7] Schmidtke DW, Diamond SL (2000) Direct Observation of Membrane Tethers Formed during Neutrophil Attachment to Platelets or P-Selectin under Physiological Flow. J Cell Biol 149: 719–730. 10791984 10.1083/jcb.149.3.719 PMC2174847

[8] Liu AP, Richmond DL, Maibaum L, Pronk S, Geissler PL, et al (2008) Membrane-induced bundling of actin filaments. Nat Phys 4: 789–793. 19746192 10.1038/nphys1071 PMC2739388

[9] Enculescu M, Sabouri-Ghomi M, Danuser G, Falcke M (2010) Modeling of Protrusion Phenotypes Driven by the Actin-Membrane Interaction. Biophys J 98: 1571–1581. 20409477 10.1016/j.bpj.2009.12.4311 PMC2856167

[10] Yang C, Hoelzle M, Disanza A, Scita G, Svitkina T (2009) Coordination of Membrane and Actin Cytoskeleton Dynamics during Filopodia Protrusion. PLoS ONE 4: e5678. 19479071 10.1371/journal.pone.0005678 PMC2682576

[11] Schwan C, Nölke T, Kruppke AS, Schubert DM, Lang AE, et al (2011) Cholesterol- and Sphingolipid-rich Microdomains Are Essential for Microtubule-based Membrane Protrusions Induced by Clostridium difficile Transferase (CDT). J Biol Chem 286: 29356–29365. 21705797 10.1074/jbc.M111.261925 PMC3190741

[12] Zhang X, Hurng J, Rateri DL, Daugherty A, Schmid-Schönbein GW, et al (2011) Membrane cholesterol modulates the fluid shear stress response of polymorphonuclear leukocytes via its effects on membrane fluidity. Am J Physiol - Cell Physiol 301: C451–C460. 21525434 10.1152/ajpcell.00458.2010 PMC3154559

[13] Khatibzadeh N, Gupta S, Farrell B, Brownell WE, Anvari B (2012) Effects of cholesterol on nano-mechanical properties of the living cell plasma membrane. Soft Matter 8: 8350–8360. 23227105 10.1039/C2SM25263E PMC3515074

[14] Zidovetzki R, Levitan I (2007) Use of cyclodextrins to manipulate plasma membrane cholesterol content: Evidence, misconceptions and control strategies. Biochim Biophys Acta (BBA) - Biomembranes 1768: 1311–1324. 17493580 10.1016/j.bbamem.2007.03.026 PMC1948080

[15] Rajagopalan L, Greeson JN, Xia A, Liu H, Sturm A, et al (2007) Tuning of the Outer Hair Cell Motor by Membrane Cholesterol. J Biol Chem 282: 36659–36670. 17933870 10.1074/jbc.M705078200 PMC2679373

[16] Sfondouris J, Rajagopalan L, Pereira FA, Brownell WE (2008) Membrane Composition Modulates Prestin-associated Charge Movement. J Biol Chem 283: 22473–22481. 18567583 10.1074/jbc.M803722200 PMC2504877

[17] Coué M, Brenner SL, Spector I, Korn ED (1987) Inhibition of actin polymerization by latrunculin A. FEBS Lett 213: 316–318. 3556584 10.1016/0014-5793(87)81513-2

[18] Morton WM, Ayscough KR, McLaughlin PJ (2000) Latrunculin alters the actin-monomer subunit interface to prevent polymerization. Nat Cell Biol 2: 376–378. 10854330 10.1038/35014075

[19] Sun M, Graham JS, Hegedüs B, Marga F, Zhang Y, et al (2005) Multiple Membrane Tethers Probed by Atomic Force Microscopy. Biophys J 89: 4320–4329. 16183875 10.1529/biophysj.104.058180 PMC1366996

[20] Li Z, Anvari B, Takashima M, Brecht P, Torres JH, et al (2002) Membrane Tether Formation from Outer Hair Cells with Optical Tweezers. Biophys J 82: 1386–1395. 11867454 10.1016/S0006-3495(02)75493-3 PMC1301940

[21] Ermilov SA, Murdock DR, Qian F, Brownell WE, Anvari B (2007) Studies of plasma membrane mechanics and plasma membrane–cytoskeleton interactions using optical tweezers and fluorescence imaging. J Biomech 40: 476–480. 16500663 10.1016/j.jbiomech.2005.12.006

[22] Qian F, Ermilov S, Murdock D, Brownell WE, Anvari B (2004) Combining optical tweezers and patch clamp for studies of cell membrane electromechanics. Rev Sci Instrum 75: 2937–2942. 21412445 10.1063/1.1781382 PMC3056459

[23] Lim CT, Zhou EH, Quek ST (2006) Mechanical models for living cells—a review. J Biomech 39: 195–216. 16321622 10.1016/j.jbiomech.2004.12.008

[24] Schmitz J, Benoit M, Gottschalk K-E (2008) The Viscoelasticity of Membrane Tethers and Its Importance for Cell Adhesion. Biophys J 95: 1448–1459. 18456832 10.1529/biophysj.107.124289 PMC2479604

[25] YC F (1993) Biomechanics: Mechanical properties of living tissues. New York: Springer.

[26] Byfield FJ, Aranda-Espinoza H, Romanenko VG, Rothblat GH, Levitan I (2004) Cholesterol Depletion Increases Membrane Stiffness of Aortic Endothelial Cells. Biophys J 87: 3336–3343. 15347591 10.1529/biophysj.104.040634 PMC1304801

[27] Byfield FJ, Hoffman BD, Romanenko VG, Fang Y, Crocker JC, et al (2006) Evidence for the role of cell stiffness in modulation of volume-regulated anion channels. Acta Physiol 187: 285–294. 10.1111/j.1748-1716.2006.01555.x 16734765

[28] Oh H, Mohler ER, Tian A, Baumgart T, Diamond SL (2009) Membrane Cholesterol Is a Biomechanical Regulator of Neutrophil Adhesion. Arterioscler Thromb Vasc Biol 29: 1290–1297. 19667108 10.1161/ATVBAHA.109.189571 PMC2762395

[29] Evans E, Rawicz W (1990) Entropy-driven tension and bending elasticity in condensed-fluid membranes. Phys Rev Lett 64: 2094–2097. 10041575 10.1103/PhysRevLett.64.2094

[30] Gracia RS, Bezlyepkina N, Knorr RL, Lipowsky R, Dimova R (2010) Effect of cholesterol on the rigidity of saturated and unsaturated membranes: fluctuation and electrodeformation analysis of giant vesicles. Soft Matter 6: 1472–1482.

[31] Pan J, Mills TT, Tristram-Nagle S, Nagle JF (2008) Cholesterol Perturbs Lipid Bilayers Nonuniversally. Phys Rev Lett 100: 198103. 18518492 10.1103/PhysRevLett.100.198103 PMC2695669

[32] Pan J, Tristram-Nagle S, Nagle JF (2009) Effect of cholesterol on structural and mechanical properties of membranes depends on lipid chain saturation. Phys Rev E 80: 021931. 10.1103/PhysRevE.80.021931 PMC2756665 19792175

[33] Needham D, Nunn RS (1990) Elastic deformation and failure of lipid bilayer membranes containing cholesterol. Biophys J 58: 997–1009. 2249000 10.1016/S0006-3495(90)82444-9 PMC1281045

[34] Liu AP, Fletcher DA (2006) Actin Polymerization Serves as a Membrane Domain Switch in Model Lipid Bilayers. Biophys J 91: 4064–4070. 16963509 10.1529/biophysj.106.090852 PMC1635687

[35] Lulevich V, Yang H-y, Rivkah Isseroff R, Liu G-y (2010) Single cell mechanics of keratinocyte cells. Ultramicroscopy 110: 1435–1442. 20728993 10.1016/j.ultramic.2010.07.009

[36] Spector I, Shochet N, Kashman Y, Groweiss A (1983) Latrunculins: novel marine toxins that disrupt microfilament organization in cultured cells. Science 219: 493–495. 6681676 10.1126/science.6681676

[37] Borghi N, Brochard-Wyart F (2007) Tether Extrusion from Red Blood Cells: Integral Proteins Unbinding from Cytoskeleton. Biophys J 93: 1369–1379. 17526591 10.1529/biophysj.106.087908 PMC1929048

[38] Hochmuth FM, Shao JY, Dai J, Sheetz MP (1996) Deformation and flow of membrane into tethers extracted from neuronal growth cones. Biophys J 70: 358–369. 8770212 10.1016/S0006-3495(96)79577-2 PMC1224934

[39] Marcus W, Hochmuth R (2002) Experimental Studies of Membrane Tethers Formed from Human Neutrophils. Ann Biomed Eng 30: 1273–1280. 12540203 10.1114/1.1528614

[40] Waugh R, Bauserman R (1995) Physical measurements of bilayer-skeletal separation forces. Ann Biomed Eng 23: 308–321. 7631984 10.1007/BF02584431

[41] Xu G, Shao J-Y (2008) Human neutrophil surface protrusion under a point load: location independence and viscoelasticity. Am J Physiol - Cell Physiol 295: C1434–C1444. 18815230 10.1152/ajpcell.00136.2008 PMC2584998

[42] Marion S, Wilhelm C, Voigt H, Bacri J-C, Guillén N (2004) Overexpression of myosin IB in living Entamoeba histolytica enhances cytoplasm viscosity and reduces phagocytosis. J Cell Sci 117: 3271–3279. 15226399 10.1242/jcs.01178

[43] Tsai MA, Frank RS, Waugh RE (1994) Passive mechanical behavior of human neutrophils: effect of cytochalasin B. Biophys J 66: 2166–2172. 8075350 10.1016/S0006-3495(94)81012-4 PMC1275942

[44] Chen Y, Girdhar G, Shao J-Y (2007) Single membrane tether extraction from adult and neonatal dermal microvascular endothelial cells. Am J Physiol - Cell Physiol 292: C1272–C1279. 17079334 10.1152/ajpcell.00251.2006

[45] Titushkin I, Cho M (2006) Distinct Membrane Mechanical Properties of Human Mesenchymal Stem Cells Determined Using Laser Optical Tweezers. Biophys J 90: 2582–2591. 16399828 10.1529/biophysj.105.073775 PMC1403190

[46] Murdock DR, Ermilov SA, Spector AA, Popel AS, Brownell WE, et al (2005) Effects of Chlorpromazine on Mechanical Properties of the Outer Hair Cell Plasma Membrane. Biophys J 89: 4090–4095. 16199506 10.1529/biophysj.105.069872 PMC1366974

[47] Fabry B, Maksym GN, Butler JP, Glogauer M, Navajas D, et al (2003) Time scale and other invariants of integrative mechanical behavior in living cells. Phys Rev E 68: 041914. 10.1103/PhysRevE.68.041914 14682980

[48] Roy S, Brownell WE, Spector AA (2012) Modeling Electrically Active Viscoelastic Membranes. PLoS ONE 7: e37667. 22701528 10.1371/journal.pone.0037667 PMC3365126

[49] Stamenović D, Rosenblatt N, Montoya-Zavala M, Matthews BD, Hu S, et al (2007) Rheological Behavior of Living Cells Is Timescale-Dependent. Biophys J 93: L39–L41. 17693464 10.1529/biophysj.107.116582 PMC1989695

[50] Zhou EH, Xu F, Quek ST, Lim CT (2012) A power-law rheology-based finite element model for single cell deformation. Biomech Model Mechanobiol 11: 1075–1084. 22307682 10.1007/s10237-012-0374-y

