# Supplementary figures and images for: Effects of Plasma Membrane Cholesterol Level and Cytoskeleton F-Actin on Cell Protrusion Mechanics
Source: PLoS One. 2013 Feb 22;8(2):e57147. doi: 10.1371/journal.pone.0057147 (PMC3579816; doi:10.1371/journal.pone.0057147)

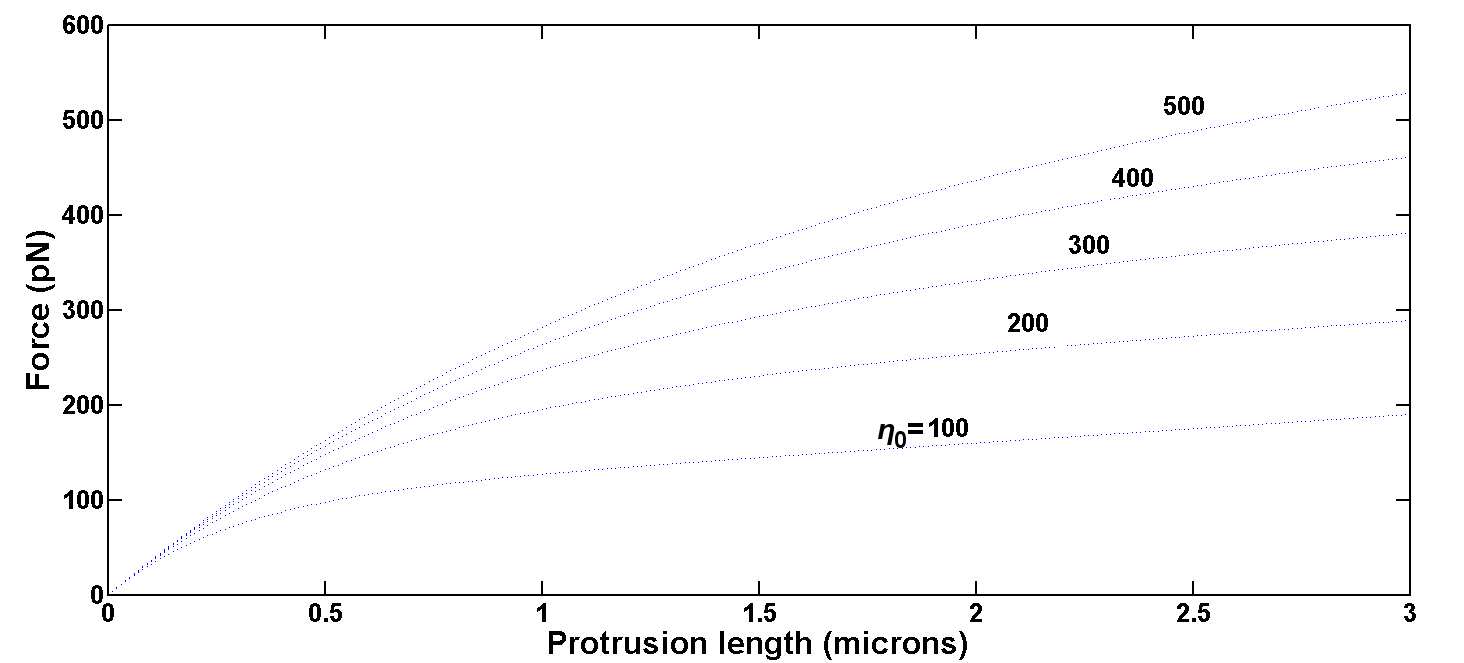

Supplement: Figure S3 — Effects of changes in η0 on protrusion force-length plots based on SLS model. Values of parameters k 0 and k 1 are kept constant at 350 pN/µm and 30 pN/µm, respectively, while η0 (pN.s/µm) changes as shown. The SLS model predicts formation of longer protrusion associated with lower values of viscosity in response to a given force value. Effects of the viscous parameter become more pronounced at the later stages of protrusion formation. (TIF) [file pone.0057147.s003.tif]

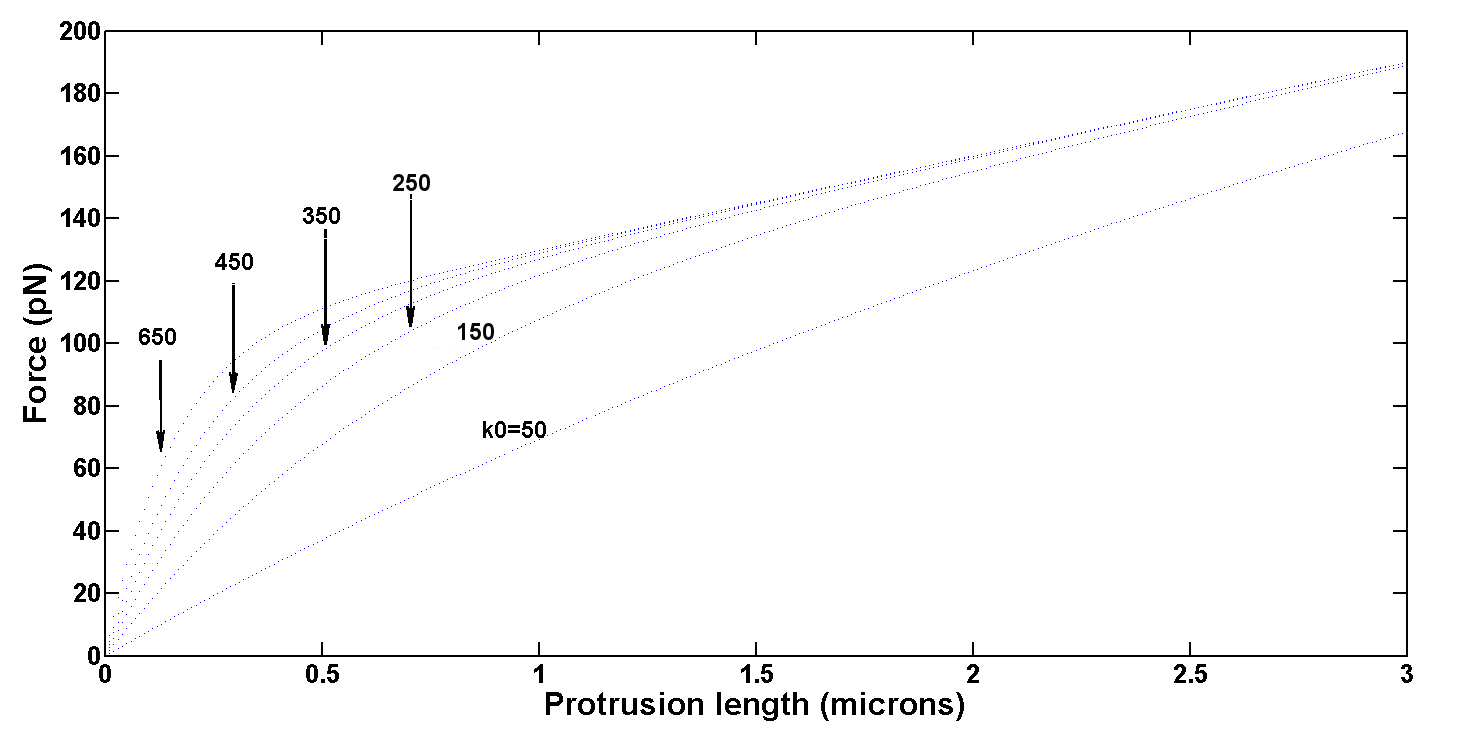

Supplement: Figure S4 — Effects of changes in k 0 on protrusion force-length plots based on SLS model. Values of parameters k 1 and η0 are kept constant at 30 pN/µm and 100 pN.s/µm, respectively, while k 0 (pN) changes as shown. The profiles indicate the effects of the k 0 stiffness at the early stage of the protrusion formation, and formation of shorter protrusion under a given force value in response to higher values of k 0 stiffness. (TIF) [file pone.0057147.s004.tif]

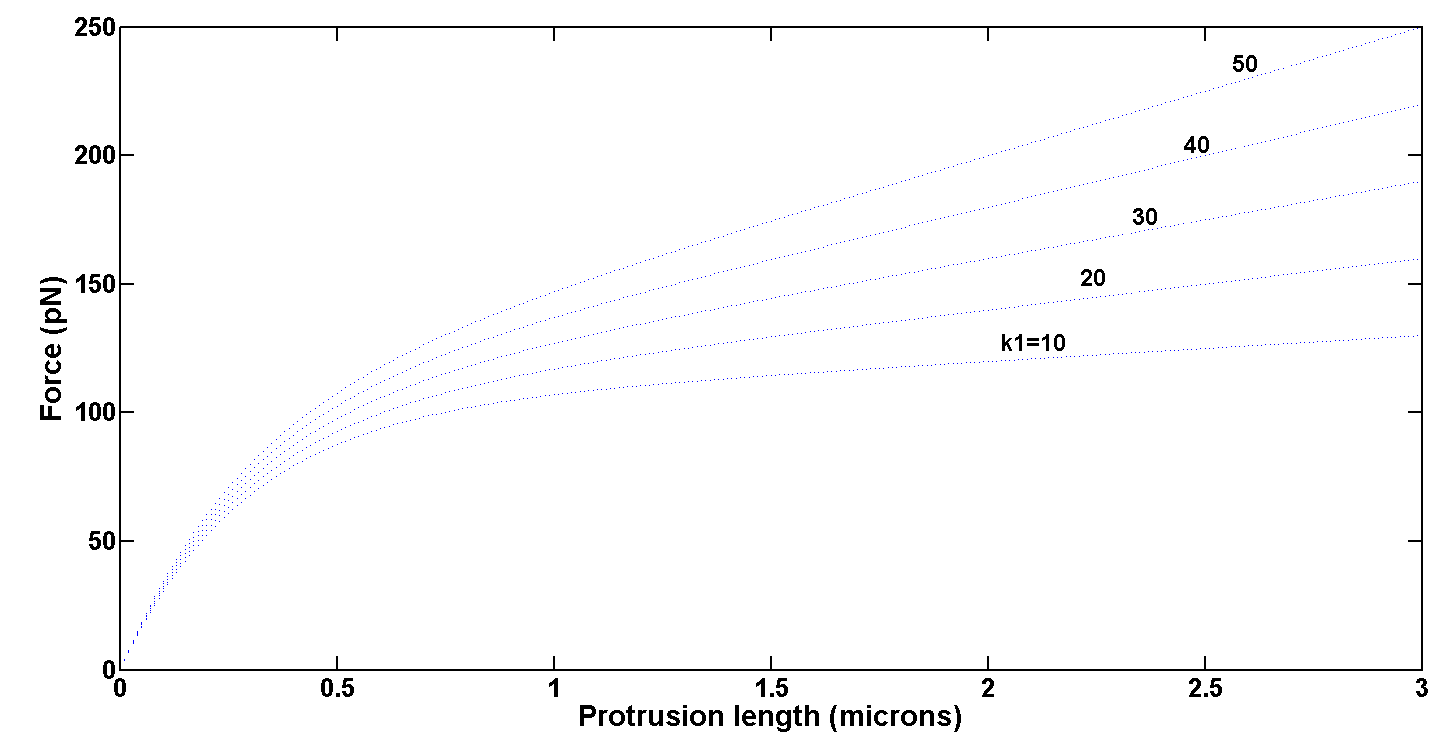

Supplement: Figure S5 — Effects of changes in k 1 on protrusion force-length plots based on SLS model. Values of parameters k 0 and η0 are kept constant at 350 pN/µm and 100 pN.s/µm, respectively while k 1 (pN) changes as shown. The k 1 stiffness affects the late stage of the protrusion formation. Higher values of k 1 are associated with formation of shorter protrusions under a given force value. (TIF) [file pone.0057147.s005.tif]
