# Supplementary material for: Effects of Plasma Membrane Cholesterol Level and Cytoskeleton F-Actin on Cell Protrusion Mechanics
Source: PLoS One. 2013 Feb 22;8(2):e57147. doi: 10.1371/journal.pone.0057147 (PMC3579816; doi:10.1371/journal.pone.0057147)
Supplement: Text S1 — Imaging the cell protrusion formation. (DOC) [file pone.0057147.s006.doc]

**Imaging the cell protrusion formation**

Protrusions were formed from human embryonic kidney (HEK 293) cells adhered to the glass bottom of petri dishes pre-coated with poly-D-lysine to enhance adhesion and growth of the cells over the substrate surface. A sequence of images showing the protrusion formation process over ≈ 6 seconds time interval is shown in Fig. S1. Images were obtained using bright-field microscopy (white-light illumination). A microsphere (bead) was trapped with laser tweezers and then brought into proximity of an adherent cell to establish contact between the bead and the cell. After 10-15 seconds of contact time to achieve bead-plasma membrane adhesion, the cell was moved away from the trapped bead at 1 µm/sec with the piezoelectric translation stage, resulting in application of a tensile force over the cell surface and subsequent formation of a conically-shaped protrusion at the cell surface.

The image sequence shows a transition from a conically-shaped cell surface protrusion (images 1-10) to a tubular shaped tether in response to further elongation (images 12-15). This transition indicates the separation of the plasma membrane from the cytoskeleton, and is associated with a sudden drop in the force value in protrusion force profiles (Fig. S2). Such force profiles have been previously reported during tether formation experiments . In this study, we focused on the cell surface protrusions prior to its transition into a tether.

**References**

1. Ermilov SA, Murdock DR, El-Daye D, Brownell WE, Anvari B (2005) Effects of Salicylate on Plasma Membrane Mechanics. J Neurophysiol 94: 2105-2110.

2. Ermilov SA, Murdock DR, Qian F, Brownell WE, Anvari B (2007) Studies of plasma membrane mechanics and plasma membrane–cytoskeleton interactions using optical tweezers and fluorescence imaging. J Biomech 40: 476-480.

3. Khatibzadeh N, Gupta S, Farrell B, Brownell WE, Anvari B (2012) Effects of cholesterol on nano-mechanical properties of the living cell plasma membrane. Soft Matter 8: 8350-8360.

4. Li Z, Anvari B, Takashima M, Brecht P, Torres JH, et al. (2002) Membrane Tether Formation from Outer Hair Cells with Optical Tweezers. Biophys J 82: 1386-1395.

5. Pontes B, Viana NB, Salgado LT, Farina M, Neto VM, et al. (2011) Cell Cytoskeleton and Tether Extraction. Biophys J 101: 43-52.
