# Supplementary material for: Effects of Plasma Membrane Cholesterol Level and Cytoskeleton F-Actin on Cell Protrusion Mechanics
Source: PLoS One. 2013 Feb 22;8(2):e57147. doi: 10.1371/journal.pone.0057147 (PMC3579816; doi:10.1371/journal.pone.0057147)
Supplement: Text S2 — Illustrative dynamic force profile. (DOC) [file pone.0057147.s007.doc]

**Illustrative dynamic force profile**

Application of a tensile pulling force over a patch area at the surface of adherent cells resulted in formation of cellular protrusion. During the first stage of the pulling process (A-B) (Fig. S2), the force increases from zero at the initiation of the pulling process (A), and reaches the first maximum value (B). Point B in the force plot is associated with (*F*_max_, *l*_pt-max_) in our analyses. The peak force at B is followed by a sudden and identifiable drop in the force value to point C. The drop in the force value at point B indicates the separation of the plasma membrane from the underlying cytoskeleton, and is associated with a transition from conical shaped protrusions to tubular (tether) structure (Fig. S1). As pulling of the tether continues to a desired length, the force increases to a second peak (Point D) at the end of the pulling process where the tether length is held constant.

The force starts to relax at point D under constant tether length. The tether force continues to relax till reaching an equilibrium value at point E. The tether ruptures at point E, causing a rapid return of the trapped bead to the trap center (point F). During the interval F-G, the force value is at zero, indicating the absence of any external forces on the bead (trapping force remains the only force during this interval).

The tether force profile shown in Fig. S2 is representative, and resembles other such profiles as reported in our previous publications . For example, the respective mean and standard deviation values of the peak force at point B for protrusions formed at the rate of 1 µm/s from normal HEK cells,  based on  sample size of n=15, are  246.3 pN, and 87.6 pN .

**References**

1. Ermilov SA, Murdock DR, El-Daye D, Brownell WE, Anvari B (2005) Effects of Salicylate on Plasma Membrane Mechanics. J Neurophysiol 94: 2105-2110.

2. Ermilov SA, Murdock DR, Qian F, Brownell WE, Anvari B (2007) Studies of plasma membrane mechanics and plasma membrane–cytoskeleton interactions using optical tweezers and fluorescence imaging. J Biomech 40: 476-480.

3. Khatibzadeh N, Gupta S, Farrell B, Brownell WE, Anvari B (2012) Effects of cholesterol on nano-mechanical properties of the living cell plasma membrane. Soft Matter 8: 8350-8360.

4. Li Z, Anvari B, Takashima M, Brecht P, Torres JH, et al. (2002) Membrane Tether Formation from Outer Hair Cells with Optical Tweezers. Biophys J 82: 1386-1395.

5. Qian F, Ermilov S, Murdock D, Brownell WE, Anvari B (2004) Combining optical tweezers and patch clamp for studies of cell membrane electromechanics. Rev Sci Instrum 75: 2937-2942.
