# Supplementary material for: Effects of Plasma Membrane Cholesterol Level and Cytoskeleton F-Actin on Cell Protrusion Mechanics
Source: PLoS One. 2013 Feb 22;8(2):e57147. doi: 10.1371/journal.pone.0057147 (PMC3579816; doi:10.1371/journal.pone.0057147)
Supplement: Text S3 — Parametric analysis. (DOC) [file pone.0057147.s008.doc]

**Parametric analysis**

We performed a parametric analysis to investigate the effects of *η_0_*, *k*_0_, and *k*_1_ of the SLS model on the resulting force-protrusion length profiles (Figures S3-S5). In Fig. S3, we present the effects of the viscous parameter (*η*_0_) of the Maxwell body on protrusions, under constant values of the Maxwell stiffness (*k*_0_) and the stiffness of the parallel spring (*k*_1_). The SLS model predicts formation of longer protrusion associated with lower values of viscosity in response to a given force value. Effects of the viscous parameter become more pronounced at the later stages of protrusion formation.

Figure S4 shows the effects of changes in the stiffness of the Maxwell body (*k*_0_) on protrusion force-displacement plots, under constant values of viscosity (*η*_0_) and spring constant (*k*_1_). The profiles indicate the effects of the *k*_0_ stiffness at the early stage of the protrusion formation, and formation of shorter protrusion under a given force value in response to higher values of *k*_0_ stiffness.

Effects of changes in values of the *k*_1_ stiffness on force-length plots predicted by the SLS model, under constant values of the Maxwell elements *k*_0_, and *η*_0_, are shown in Figure S5. The *k*_1_ stiffness affects the late stage of the protrusion formation. Higher values of *k*_1_ are associated with formation of shorter protrusions under a given force value.
